# Association between serum sphingolipids and necroinflammation of liver tissue pathology in chronic hepatitis B

**DOI:** 10.7150/ijms.75820

**Published:** 2022-11-21

**Authors:** Yan Ren, Jing Zhao, Manman Xu, Yang Wang, Li Bai, Yingying Jiang, Shuang Liu, Yu Chen, Zhongping Duan, Sujun Zheng

**Affiliations:** 1 Liver Disease Center, Beijing YouAn Hospital, Capital Medical University, Beijing China 100069.; 2 Beijing Municipal Key Laboratory of Liver Failure and Artificial Liver Treatment & Research, Beijing YouAn Hospital, Capital Medical University, Beijing China 100069.

**Keywords:** sphingolipid, Ceramide, liver necroinflammation, chronic hepatitis B

## Abstract

**Background & Aims:** Accurately identifying liver necroinflammation was essential for the timely implementation of antiviral therapy in chronic hepatitis B(CHB) patients. The sphingolipids were involved in various chronic inflammatory processes. This study aimed to evaluate the association between serum sphingolipids and liver necroinflammation in CHB patients.

**Methods:** The study prospectively enrolled patients with a diagnosis of chronic hepatitis B who were subsequently treated with nucleos(t)ide analogs (NAs). Liver biopsy was performed at baseline and 5-year follow-up, and serum sphingolipid levels were measured by ultra-high-performance liquid chromatography tandem mass spectrometry.

**Results:** A total of 70 CHB patients were enrolled with baseline liver necroinflammation of 27(38.6%) G1, 23(32.9%) G2, and 20(28.6%) G ≥ 3, respectively. A total of 126 liver biopsies were performed on the study population over a 5-year period, of which 80 (63.5%) G<2 and 46 (36.5%) G≥2. Serum ALT, ALP, SM d16:0/16:1, SM d16:0/17:1, SM d18:0/17:0 and Cer d18:2/22:0 showed significant differences between two groups (P<0.01). Multivariate analysis showed that serum ALT (OR 1.006, 95% CI: 1.000-1.011), SM d16:0/16:1 (OR 1.552, 95% CI: 1.150-2.093), Cer d18:2/22:0 (OR 0.003, 95% CI: 0.000-0.173) were associated with G ≥ 2. In the subgroup of patients with normal serum ALT, serum Cer d18:2/22:0 was lower in patients with G ≥ 2 than that with G < 2. After 5 years, alleviated inflammation was accompanied by decreased serum SM d16:0/16:1 and increased serum Cer d18:2/22:0 in patients with baseline G ≥ 2.

**Conclusions:** Lower serum Cer d18:2/22:0 could reflect hepatic necroinflammation (G ≥ 2) in CHB patients including those with normal serum ALT, and its elevation predicts the inflammation improvement after NAs treatment.

## Introduction

Chronic hepatitis B (CHB) is a global public problem, and effective antiviral treatments can significantly reduce the occurrence of hepatitis B-related cirrhosis and liver cancer 1. If the liver biopsy shows moderate or severe necroinflammation or significant fibrosis, initiating antiviral therapy is recommended 2. Therefore, timely and accurate identification of the grading of hepatic necroinflammation is important for the initiation of antiviral therapy. Liver biopsy is the gold standard for the evaluation of fibrosis and necroinflammation 2. Due to the risk of serious complications, its application is limited 3. Alanine aminotransferase (ALT) is the most commonly noninvasive biomarker reflecting liver necroinflammation. However, some studies showed that CHB patients with severe liver inflammation exhibited normal levels of serum ALT 4. In this way, serum ALT does not always reflect the degree of liver necroinflammation. Noninvasive hepatic necroinflammatory biomarkers are urgently needed.

Sphingolipids play a role in the stability of the cell membrane as well as in signal transduction pathways involved in inflammatory diseases 5, 6. Ceramides, as the center of sphingolipid metabolism 7, had been suggested to be a novel biomarker in inflammatory diseases, such as rheumatoid arthritis, multiple sclerosis, inflammatory bowel disease 8, 9. Sphingolipids are also associated with inflammatory processes in liver disease. Serum sphingomyelin can reflect hepatic injury in patients with CHB 10. In NASH, pro-inflammatory cytokines are significantly correlated with ceramides and that can influence disease severity 11, 12. There was an association between sphingomyelin and hepatic inflammation in chronic hepatitis C patients 13.

Based on the related research of sphingolipids and liver diseases, we hypothesized that sphingolipids may act as mediators in response to liver inflammation in CHB patients. In this study, we hope to screen out serum sphingolipids that can accurately reflect liver inflammation.

## Methods

### Patients

This study screened CHB patients who visited the Beijing YouAn Hospital from June 2007 to July 2008. The enrolled patients were aged ≥16 years and met the diagnostic criteria of the American Association for the Study of Liver Diseases (AASLD) for CHB. Patients with any of the following should be excluded: (1) Co-infection with hepatitis C virus, Epstein-Barr virus, cytomegalovirus, human immunodeficiency virus, or the existence of immune and alcohol-related liver disease; (2) Decompensated cirrhosis; (3) Uncontrolled severe heart, kidney disease or other organ diseases; (4) History of malignant tumors, including carcinoma in situ and atypical hyperplastic nodules;(5) Mental illness;(6) Received corticosteroids, immunosuppressive, and chemotherapy drugs within 6 months before enrollment; (7) Pregnant and breast-feeding women. A total of 70 CHB patients were included. 39 (55.71%) patients received entecavir 0.5 mg once daily, and the other 31 (44.29%) patients received adefovir 10 mg once daily for antiviral therapy.

The study was approved by the Ethics Committee of Beijing YouAn Hospital, Capital Medical University by the Helsinki Declaration, and each patient signed informed consent.

### Clinical and laboratory assessments

The patients' epidemiological and clinical data were collected at baseline and 5 years follow-up, including gender, age, Serum ALT, total bilirubin (TBIL), direct bilirubin (DBIL), gamma-glutamyl transferase (GGT), alkaline phosphatase (ALP). The liver function indexes were determined by Olympus Au5400 automatic biochemistry analyzer (Olympus, Tokyo, Japan).

### Histological evaluation

Ultrasound-guided liver biopsy was performed baseline and 5 years follow-up. A minimal 18mm length of liver tissue containing at least 12 portal areas was obtained for pathological evaluation. All sections were stained with hematoxylin and eosin. The same experienced pathologist evaluated the sections for grading necroinflammation (G) using the Scheuer scoring system. G≥2 is defined as significant necroinflammation.

### Measurement of serum sphingolipids

Patients' fasting serum was collected at the time point of two liver biopsies and stored in a -80℃ refrigerator for subsequent detection of sphingolipids. A total of 39 serum sphingolipids were tested, including 32 serum sphingomyelins and 7 serum ceramides. Sphingolipids were measured by ultra-high-performance liquid chromatography tandem mass spectrometry (UHPLC-MS/MS). UHPLC-MS/MS was performed on an AB SCIEX Triple QuadTM 5500 mass spectrometer (QQQ: AB SCIEX, Boston, USA) containing a triple quadrupole MS analyzer with an electrospray ionization interface and an Shimazu LC-20AXR system (LC: Shimazu, Kyoto, Japan). The lipidomics minimal reporting checklist and detailed experimental procedures were provided in the supplementary materials.

### Statistical analysis

All data were analyzed using IBM SPSS26.0 and GraphPad Prism Version 8.0 (GraphPad Software, La Jolla, CA, USA). Continuous variables were presented as mean ± standard deviation (SD) or median (inter-quartile range, IQR). Categorical variables were presented as n (%). Mann-Whitney U test was used to compare variables among different hepatic necroinflammation groups. Wilcoxon test was applied to compare paired samples. Clinical characteristics associated with necroinflammation(G≥2) in CHB patients were assessed using logistic regression analysis, which were fitted with a stepwise method using significant factors (P<0.01) that had been prefiltered in univariate regression analysis to identify the independent relationship between sphingolipids and necroinflammation(G≥2). The diagnostic values for the examined markers were calculated using the receiver operating characteristic (ROC) and the area under the curve (AUC). P<0.05 was considered statistically significant.

## Results

### Patient characteristics

**Figure 1:**
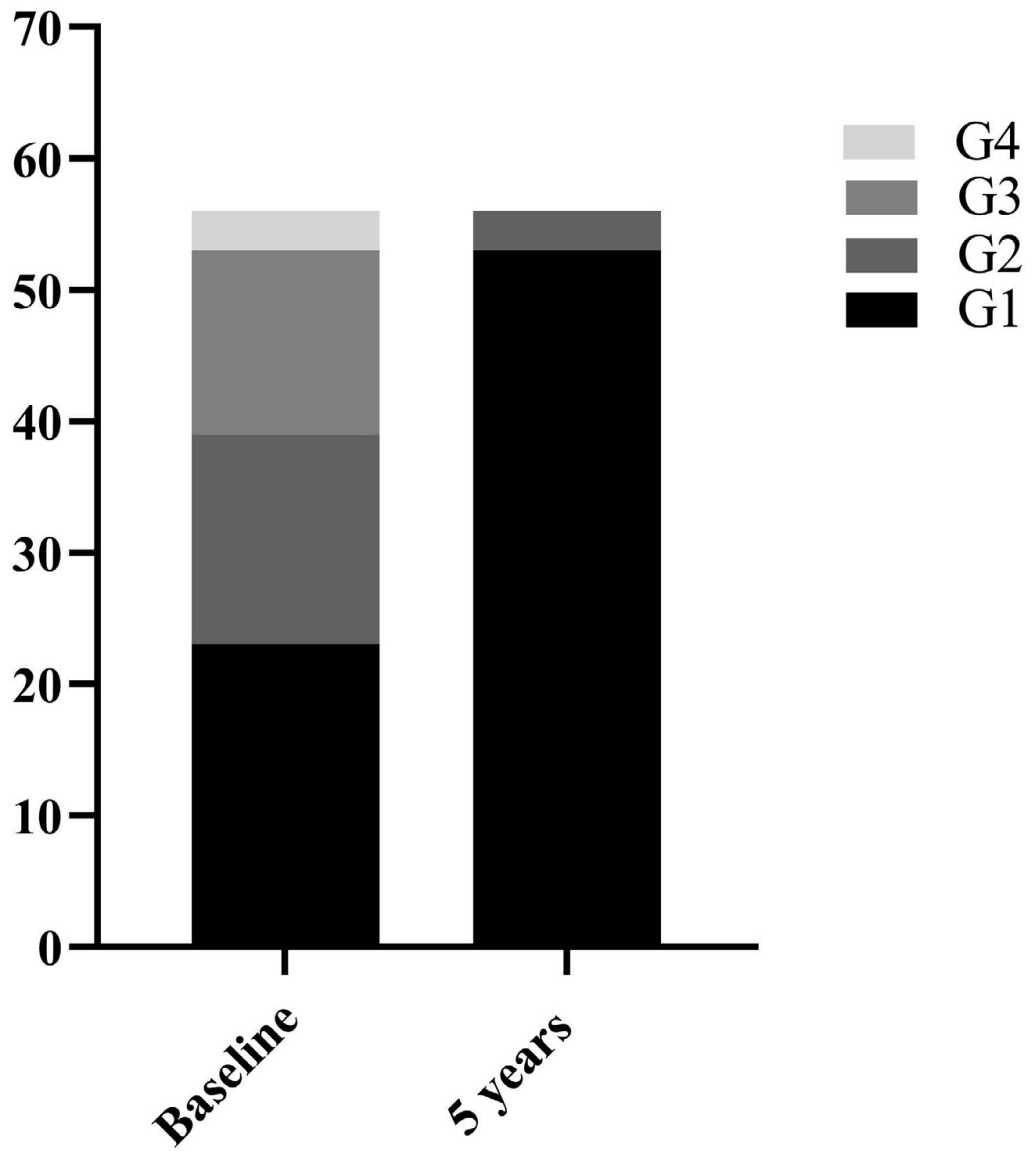
56 CHB patients with liver necroinflammation composition ratio at baseline and 5 years follow-up.

Among the 70 CHB patients, 58 were male and 12 were female with a mean age of 36.6 ± 9.32 years. All patients underwent liver biopsy at baseline. Based on histopathological manifestations of the liver, 27(38.6%) patients were classified as mild necroinflammation (G=1), 23(32.9%) as moderate necroinflammation (G=2), and 20(28.6%) as severe necroinflammation (G≥3). 56 of the 70 CHB patients underwent liver biopsy again after 5 years of follow-up. The clinical characteristics of 70 CHB patients at baseline and 56 at 5 years of follow-up were shown in Table 1. Among the 56 CHB patients, the number of patients with G≥2 decreased from 33 (58.9%) at baseline to 3 (5.4%) after 5 years of follow-up (Figure 1). The comparison of clinical indicators between G<2 and G≥2 groups in 126 cases of liver biopsies were shown in Table 1. Median serum ALT 25.10 (17.70-61.10) U/L in G<2 group was significantly lower than 71.60(40.97-135.30) U/L in G≥2 group (P<0.001).

### Serum sphingolipids and necroinflammation(G≥2) in CHB patients

In 70 CHB patients, serum SM d16:0/16:1 and Cer d18:2/22:0 of 39 serum sphingolipids at baseline were significantly different between G<2 and G≥2 groups. A total of 126 liver biopsies, including 80 G<2 and 46 G≥2 over 5 years were further analyzed. The 11 sphingolipids including serum SM d16:0/16:1 and Cer d18:2/22:0 were significantly different between the two groups (P<0.05). Serum SM d16:0/16:1, SM d16:0/17:1, SM d18:0/17:0 and Cer d18:2/22:0 were 4.77±1.41 μmol/L vs 5.61±1.50 μmol/L, 3.62±0.96 μmol/L vs 4.12±1.12μmol/L, 1.74±0.61 μmol/L vs 2.17±0.80 μmol/L, 0.31±0.14 μmol/L vs 0.24±0.10 μmol/L in G <2 and G≥2 groups with P<0.01, respectively (Table 2). The comparison results of the other 35 serum sphingolipids were shown in Table S2.

The clinical indicators and serum sphingolipids with significant differences (P<0.01) between the two groups were included in univariate regression analysis, including serum ALT, ALP, serum SM d16:0/16:1, SM d16:0/17:1, SM d18:0/17:0 and Cer d18:2/22:0.The results demonstrated that higher levels of serum ALT, SM d16:0/16:1, SM d18:0/17:0, and lower Cer d18:2/22:0 were significantly associated with necroinflammation, but not ALP or SM d16:0/17:1 (Table 3). Further, multivariate logistic regression analysis found that serum Cer d18:2/22:0 (OR, 0.003, 95%CI 0.000-0.173) was a protective factor for the significance of liver necroinflammation. On the contrary, higher serum ALT (OR, 1.006, 95%CI 1.000-1.011), and higher serum SM d16:0/16:1 (OR, 1.552, 95%CI 1.150-2.093) were associated with increased odds of significance liver necroinflammation (P <0.05; Table 3).

We further performed ROC analyses of the predictive values of serum ALT and serum sphingolipids for significance necroinflammation and the results showed that the AUC of serum Cer d18:2/22:0 (AUC=0.621) is greater than that of serum ALT (AUC=0.549) (Figure S1).

### Serum sphingolipids and necroinflammation (G≥2) in CHB patients with normal serum ALT

**Figure 2:**
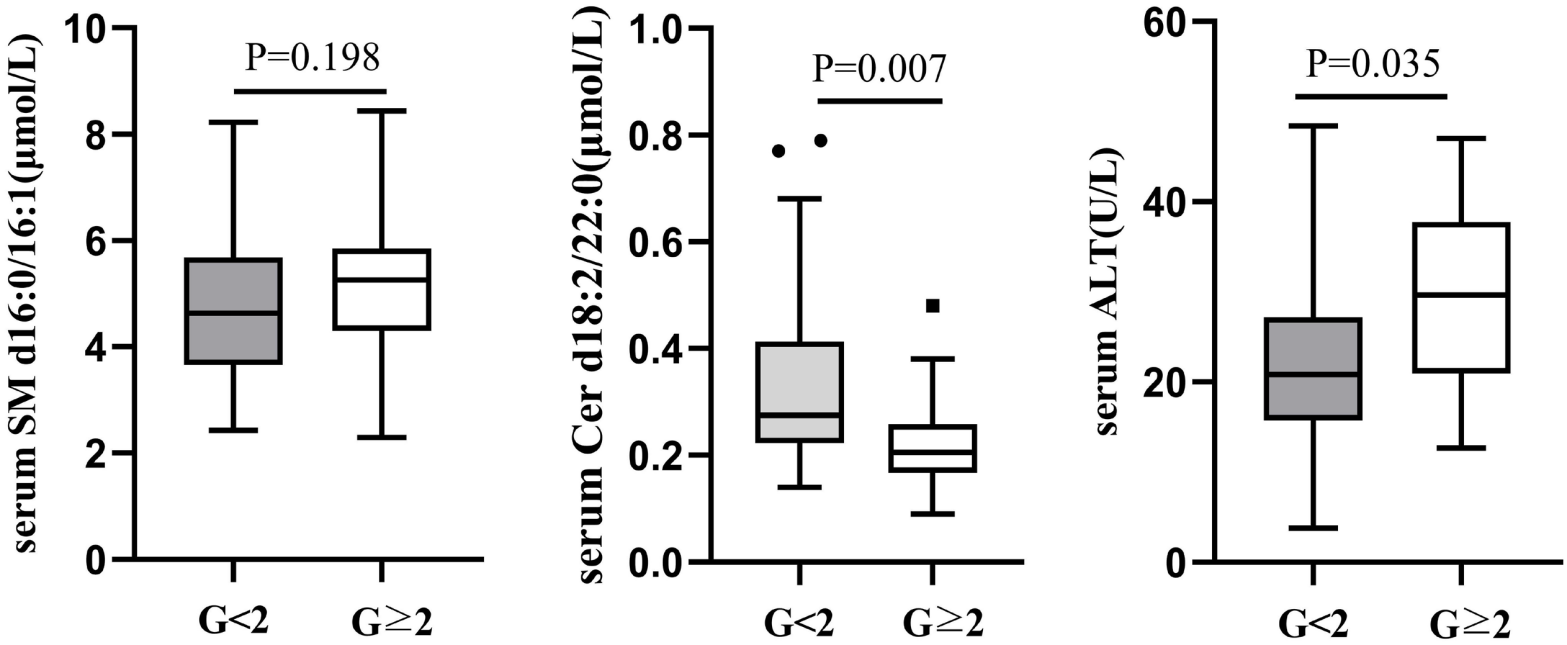
Comparison of serum SM d16:0/16:1, Cer d18:2/22:0 and ALT between G<2 and G≥2 CHB patients with normal ALT.

According to male serum ALT<50U/L and female ALT<40U/L as the normal upper line standard, the 126 liver biopsy cases were divided into normal serum ALT group and abnormal ALT group. Among 66 cases with normal ALT, 52(78.79%) had liver necroinflammation G<2 and 14 (21.21%) G≥2. There was no difference in serum SM d16:0/16:1 between the two groups (G<2 vs G≥2=4.69±1.41 μmol/L vs 5.19±1.45 μmol/L, P=0.198), whereas serum Cer d18:2/22:0 level was lower in G≥2 group (0.22±0.10μmol/L) than in G<2 (0.32±0.14μmol/L, P=0.007; Figure 2). There was still a difference in serum ALT between the G<2 and G≥2groups in 66 CHB patients with normal serum ALT (G<2 vs G≥2=22.49±9.48 U/L vs 29.56±11.25 U/L, P=0.035; Figure 2).

### Dynamic changes of serum ALT and sphingolipids in patients with baseline G≥2

**Figure 3:**
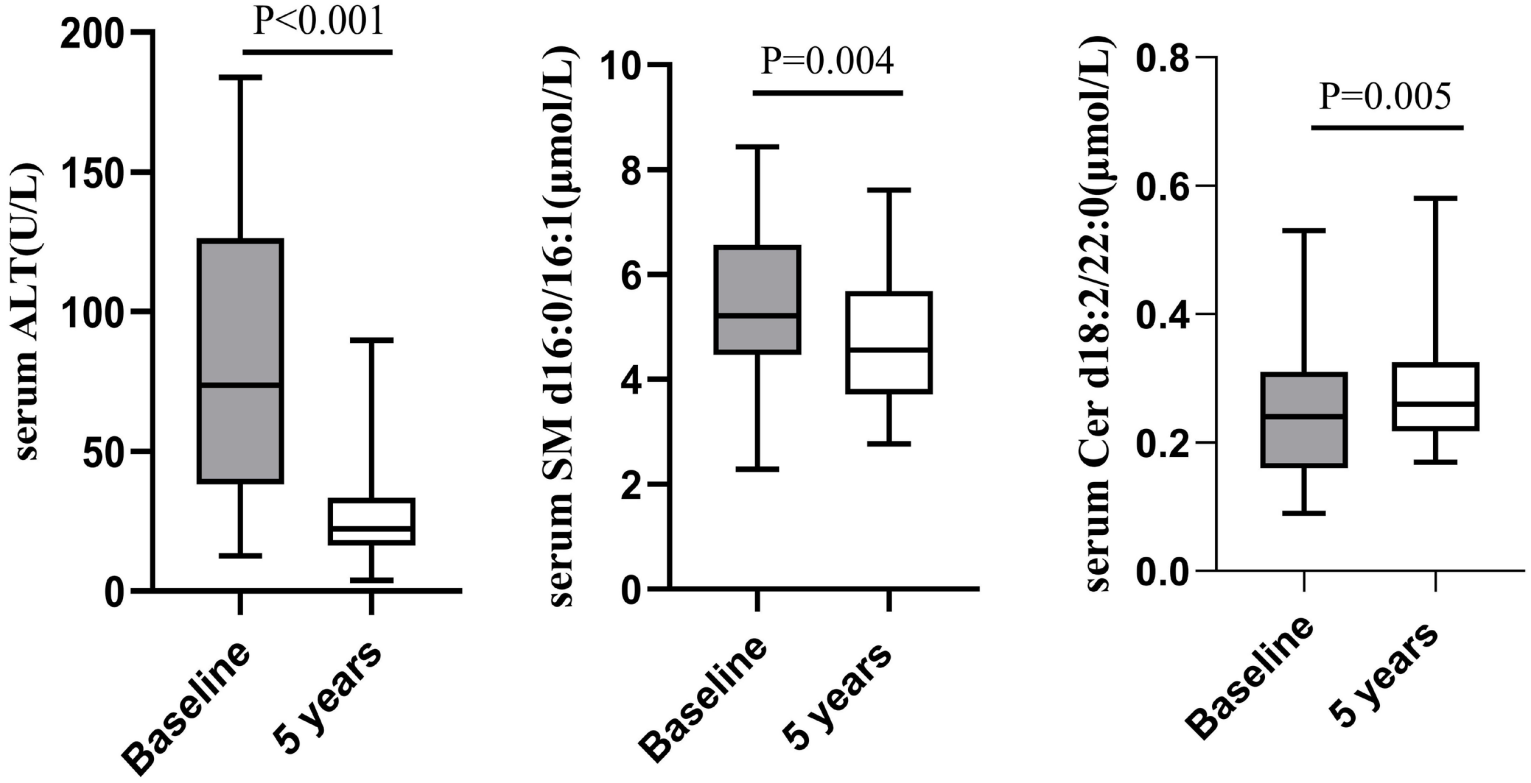
Comparison of serum ALT, SM d16:0/16:1 and Cer d18:2/22:0 between at baseline and at 5 years follow-up in patients with baseline liver necroinflammation G≥2.

There were 43 cases of liver necroinflammation G≥2 in the enrolled 70 CHB patients, of which 32 cases underwent liver biopsy again after 5 years of follow-up. The pathological results showed that except for 1 patient whose liver necroinflammation was still G2, the liver necroinflammation of the other 31 patients was alleviated (G=1). Concomitant with improvement in liver necroinflammation, the paired analysis showed that median serum ALT decreased significantly from baseline (74.00 (39.00-145.30) U/L) after 5 years of follow-up (20.00 (16.40-33.40) U/L). Compared with baseline, serum SM d16:0/16:1 decreased after 5 years follow-up (5.58±1.39 μmol/L vs 4.79±1.27 μmol/L, P=0.004), while serum Cer d18:2/22:0 increased (0.24±0.10 μmol/L vs 0.30±0.14 μmol/L, P=0.005; Figure 3).

## Discussion

In this study, we dynamically analyzed the correlation between serum sphingolipids and liver histopathological necroinflammation in patients with CHB on long-term antiviral treatment with nucleos(t)ide analogues (NAs). Lower serum Cer d18:2/22:0 correlated with significant liver necroinflammation(G≥2) even in the presence of normal ALT. With the improvement of liver necroinflammation after antiviral treatment, serum Cer d18:2/22:0 was increased. These results suggest that serum Cer d18:2/22:0 may be a potential biomarker for discriminating significant liver necroinflammation in patients with CHB.

NAs are commonly used for antiviral therapy in patients with CHB 1, 2, 14. Early initiation of antiviral therapy in patients with CHB with liver inflammation G ≥ 2 can reduce liver inflammation, fibrosis and even liver cancer 15, 16. In this study, the proportion of 56 CHB patients with liver necroinflammation G≥2 decreased from 61.43% to 5.36% after receiving long-term NAs therapy. Histopathological remission was achieved in the vast majority of CHB patients. When assessing the degree of liver necroinflammation in CHB patients, liver biopsy remains the gold standard 17. It is expected, however, that non-invasive serological indicators will replace liver biopsy due to its invasiveness and the possibility of complications. Serum ALT is currently used to reflect liver inflammation 18. Our study found higher serum ALT levels in CHB patients with G≥2. Further analysis of hepatic necroinflammation in CHB patients with normal ALT showed that 21.21% of patients had necroinflammation G≥2. This is in line with the study by Michelle et al. 19 who found that 37% of CHB patients with persistently normal ALT had significant fibrosis or inflammation. Interestingly, of the 66 cases with normal ALT, those with G≥2 still had higher ALT than those with G<2. Serum ALT <50 U/L in men and <40 U/L in women do not mean that there is no inflammatory activity in the liver. This provides a rationale for redefining the upper limit of normal ALT 20, 21.

Liver biopsy is the gold standard for reflecting liver inflammation and fibrosis, but its invasive nature, serious non-negligible complications, and the fact that it cannot be easily repeated have limited its widespread use. Our study analysed the correlation between serum sphingolipids and liver inflammation confirmed by liver biopsy and found that an elevated serum SM d16:0/16:1 and a decreased Cer d18:2/22:0 responded to significant liver inflammation. The liver not only synthesizes sphingomyelin (SM) with the choline-containing compounds absorbed from the intestine but also participates in the hydrolysis of SM due to its high sphingomyelinase (SMases) activity 22. It is therefore easy to understand the changes in serum sphingolipids when the liver is in a pathological state. The deficiency of SMases results in decreased hydrolysis of SM 23. In addition, changes in the activity of SMases can affect the levels of SM, which is linked to the occurrence and progression of chronic liver diseases 22. Previous studies found serum SM d18:1/24:0 was negatively correlated with liver necroinflammation in CHB patients 10, which seems to be inconsistent with the present report. SM with different acyl-chain lengths and saturations have different cellular functions 24. Our results showed a decrease in very-long chain SM d18:1/24:0, while an increase in long-chain SM d 16:0/16:1, was similar to studies which have shown that there was a shift from longer to shorter chains in the composition of SM in some diseases 25-28. And the C16 sphingolipids are thought to increase the susceptibility of cells to apoptosis 26. Based on the above research, we speculate that the decrease of SMase activity in HBV-infected hepatocytes leads to the accumulation of SM d 16:0/16:1, which promotes cell apoptosis and liver inflammation.

Cer d18:2/22:0 is a very long chain ceramide whose level is affected by de novo synthesis with ceramide synthase (CerS) and SM hydrolysis with sphingomyelinase 5, 29, 30. The liver is thought to be highly expressing CerS2 and CerS4 8, 9. In CerS2 null mouse, the shift of very long-chain ceramides to long-chain ceramides was found 31. In non-alcoholic fatty liver disease, the very long (C22-24)-chain ceramides exhibit a protective function 32. In addition, even ceramides with the same length of fatty acyl chains have different effects in different diseases 33, 34, which may be related to disease-specific ceramides. Whether the decrease of serum Cer d18:2/22:0 is specific to chronic hepatitis B and whether its protective effect on liver inflammation is mediated by CerS requires further studies.

Our research has limitations. Firstly, only patients with CHB were included; thus, the results cannot be generalized to other patient populations. Secondly, the combined analysis of baseline and 5-year follow-up samples may introduce bias and required validation with larger samples. Finally, the mechanism of the correlation between sphingolipids and liver necroinflammation was not further studied in this paper.

## Conclusion

To summarize, we found that lower serum Cer d18:2/22:0 was a potential noninvasive biomarker to reflect significant liver necroinflammation even with normal ALT. Alleviation of inflammation was accompanied by an increase in serum Cer d18:2/22:0.

## Supplementary Material

Supplementary information, figure and tables.

## Figures and Tables

**Table 1:** Clinical characteristics of CHB patients and comparison of clinical indicators between G < 2 and G≥2 groups

Variables	Baseline(n=70)	5 years (n=56)	Total(n=126)	G<2 (n=80)	G≥2(n=46)	*P* value
Gender (Male/female), n (%)	58(82.9)/12(17.1)	51(91.1)/5(8.9)	109(86.5)/17(13.5)	69(86.3)/11(13.8)	40(87)/6(13)	0.911
Age (yr), SD	36.6±9.32	41.46±9.57	36.53±9.39	35.07±9.45	39.08±8.83	0.013
ALT(U/L), M(IQR)	73.6(39.00-148.15)	22.30(17.10-29.40)	40.60(21.20-88.65)	25.10(17.70-61.10)	71.60(40.97-135.30)	<0.001
TBIL (µmol/L), M(IQR)	15.4(12.91-19.30)	14.05(11.07-18.40)	15.00(12.60-18.50)	14.35(12.45-18.00)	15.70(12.76-20.15)	0.266
DBIL (µmol/L), M (IQR)	4.1(2.65-6.15)	2.65(2.00-3.77)	3.20(2.20-5.00)	3.20(2.00-4.35)	4.00(2.55-6.15)	0.126
GGT (U/L), M (IQR)	37.8(17.30-82.02)	19.05(14.62-27.60)	22.70(16.32-55.15)	19.60(15.50-38.40)	32.80(16.90-66.00)	0.023
ALP (U/L), M (IQR)	83.7(73.87-114.80)	65.45(56.20-82.72)	78.70(63.55-100.90)	69.10(59.50-93.40)	87.60(72.00-115.75)	0.002
**Grade of necroinflammation, n (%)**						
G0	0(0)	0(0)	0(0)			
G1	27(38.6)	53(94.6)	80(63.5)			
G2	23(32.9)	3(5.4)	26(20.6)			
G3	17(24.3)	0(0)	17(13.5)			
G4	3(4.3)	0(0)	3(2.4)			

Abbreviations: ALT, alanine transaminase; TBIL, total bilirubin; DBIL, direct bilirubin; GGT, gamma- glutamyl transpeptidase; ALP, alkaline phosphatase; SD, standard deviation; M, median; IQR, inter-quartile range.

**Table 2:** Comparison of serum sphingolipids between G<2 and G≥2

Variables	G<2 (n=80)	G≥2 (n=46)	*P* value^✝^
SM d16:0/16:1 (μmol/L), SD	4.77±1.41	5.61±1.50	0.002
SM d16:0/17:1 (μmol/L), SD	3.62±0.96	4.12±1.12	0.008
SM d18:0/17:0 (μmol/L), SD	1.74±0.61	2.17±0.80	0.001
Cer d18:2/22:0 (μmol/L), SD	0.31±0.14	0.24±0.10	0.002

^✝^The Table showed variables with P<0.01 between the two groups.

**Table 3:** Logistic regression analysis on G≥2 of liver necroinflammation

Variables	Univariate Logistic regression analysis		Multivariate Logistic regression analysis	
	OR (95%CI)	*P* value	OR (95%CI)	*P* value
ALT	1.008 (1.002-1.013)	0.007	1.006 (1.000-1.011)	0.044
ALP	1.003 (0.997-1.009)	0.387		
SM d16:0/16:1	1.481 (1.139-1.926)	0.003	1.552 (1.150-2.093)	0.004
SM d16:0/17:1	0.921 (0.921-1.500)	0.194		
SM d18:0/17:0	2.366 (1.360-4.117)	0.002		
Cer d18:2/22:0	0.004 (0.000-0.194)	0.005	0.003 (0.000-0.173)	0.006

## Abbreviations

CHB: chronic hepatitis B
NAs: nucleos(t)ide analogs
ALT: alanine aminotransferase
TBIL: total bilirubin
DBIL: direct bilirubin
GGT: gamma-glutamyl transferase
ALP: alkaline phosphatase
UHPLC-MS/MS: Ultra-high-performance liquid chromatography tandem mass spectrometry
ROC: receiver operating characteristic
AUC: area under the curve
SMase: sphingomyelinase
CerS: ceramide synthase
