# Supplementary material for: Association between serum sphingolipids and necroinflammation of liver tissue pathology in chronic hepatitis B
Source: Int J Med Sci. 2022 Nov 21;19(14):2080–6. doi: 10.7150/ijms.75820 (PMC9724247; doi:10.7150/ijms.75820)
Supplement: Supplementary file 1 — Supplementary information, figure and tables. [file ijmsv19p2080s1.pdf]

General Lipidomics Workflow

INTERNATIONAL LIPIDOMICS SOCIETY

Overall study design

Title of the study	Association between serum sphingolipids and necroinflammation of liver tissue pathology in chronic hepatitis B		
Principle investigator	Yan Ren, Jing Zhao, Manman Xu, Yang Wang, Li Bai, Yingying Jiang, Shuang Liu, Yu Chen, Zhongping Duan, Sujun Zheng		
Institution	1 Liver Disease Center, Beijing YouAn Hospital, Capital Medical University, Beijing China 100069 2 Beijing Municipal Key Laboratory of Liver Failure and Artificial Liver Treatment & Research, Beijing YouAn Hospital, Capital Medical University, Beijing China 100069		
Corresponding Email	zhengsujun@ccmu.edu.cn		
Document creation date	10/25/2022	Clinical	Yes
Is the workflow targeted or untargeted?	Targeted		

Lipid extraction

Extraction method	2-phase system	2-phase system	MTBE
pH adjustment	None	Were internal standards added prior extraction?	Yes

Analytical platform

Number of separation dimensions	One dimension	MS type	QQQ
Separation Type 1	LC	MS vendor	SCIEX
Separation Mode 1	RP	Ion source	ESI
Separation window (1) for lipid analyte selection (±) in minutes	0.5	MS Level	MS2
RT verified by standard	Yes	Mass window for precursor ion isolation (in Da total isolation window)	1000
CCS verified by standard	No	Mass resolution for detected ion at MS2	Low resolution
Separation of isobaric/isomeric interferece confirmed	No	Resolution in Da at MS2	2400
Model for separation prediction	Yes		

Quality control

Blanks	No	Type of QC sample	Sample pool
Quality control	Yes		

Method qualification and validation

Method validation	No

Reporting

Are reported raw data uploaded into repository?	No	Raw data upload	Available on request

Sample Descriptions

Serum sphingolipids from CHB patients receiving nucleotide antiviral therapy / Human / Serum

Provided information	Freeze-thaw cycles, Storage time (month), Time to freeze (min), Time to separate plasma/serum (min)	Storage time (month)	105
Time to separate plasma/serum (min)	120	Freeze-thaw cycles	1
Temperature handling original sample	Room temperature	Additives	None
Instant sample preparation	No	Were samples stored under inert gas?	No
Time to freeze (min)	120	Additional preservation methods	No
Snap freezing in liquid N2	No	Biobank samples	No
Storage temperature	-80 °C		

Lipid Class Descriptions

Lipid class Cer[M+H]+ / Lipid identification

Lipid class	Cer	Isotope correction at MS2	No
MS Level	MS2	MS2 verified by standard	Yes
Identification level	Molecular species level	Background check at MS2	No
Polarity mode	Positive	Check isomer overlap	No
Type of positive (precursor)ion	[M+H]+	Lipid Identification Software	LipidSearch
How many fragments used for ID	2 fragments	Data manipulation	Lock mass correction
Fragment ion 1	Cer d18:1/18:0 m/z 566.6	Nomenclature for intact lipid molecule	No
Fragment ion 2	Cer d18:1/18:0 m/z 264.3	Nomenclature for fragment ions	N/A

Lipid class Cer[M+H]+ / For additional separation methods/analytical dimension

Quantitative	Yes	Limit of quantification	No
Internal lipid standard(s)	Cer 18:1;O2/25:0	Normalization to reference	No
Type of quantification	Internal standard amount	Lipid Quantification Software	Skyline
Response correction	One factor for all species	Batch correction	No
Type I isotope correction	No		

Lipid class SM[M+H]+ / Lipid identification

Lipid class	SM	Isotope correction at MS2	No
MS Level	MS2	MS2 verified by standard	Yes
Identification level	Molecular species level	Background check at MS2	No
Polarity mode	Positive	Check isomer overlap	No
Type of positive (precursor)ion	[M+H]+	Lipid Identification Software	LipidSearch
How many fragments used for ID	2 fragments	Data manipulation	Lock mass correction
Fragment ion 1	SM 32:2 m/z 673.5	Nomenclature for intact lipid molecule	No
Fragment ion 2	SM 32:2 m/z 184.1	Nomenclature for fragment ions	N/A

Lipid class SM[M+H]+ / For additional separation methods/analytical dimension

Quantitative	Yes	Limit of quantification	No
Internal lipid standard(s)	SM 18:1;O2/12:0	Normalization to reference	No
Type of quantification	Internal standard amount	Lipid Quantification Software	Skyline
Response correction	One factor for all species	Batch correction	No
Type I isotope correction	No		

Sample processing and testing of serum sphingolipids

For extraction of sphingolipids, 40 μL serum sample was added with 5 μL of the inter-standard solution (Avanti Polar Lipids, Alabaster, AL, USA) and 295 μL of methanol. Then the mixture was vortexed at room temperature for 1 hour, added with 1.0 mL methyl tert-butyl ether and thoroughly mixed. After centrifugation at 120,000 rpm for 10 minutes, the supernatant of 200 μL methyl tert-butyl ether was dried using centrifugal vacuum evaporator (Thermo Fisher Scientific, Waltham, MA, USA) for 4 hours at 4°C, then added 200 μl of isopropyl alcohol/acetonitrile with 1:1 ratio and dissolved in ultrasound. After centrifugation at 12,000 rpm for 10 minutes, 100 μL of supernatant was transferred to a 250 μl vial insert tube for testing. As stationary phase, an ACQUITY UPLC BEH C8 2.1 × 100 mm,1.8 μm column (Waters, Milford, MA, USA) was used suited for lipids retention. The mobile phase consisted of solvent A (0.1 % formic, acetonitrile and water (v:v; 6:4))and solvent B (0.1 % formic ,Isopropanol and acetonitrile (v:v;9:1)) with a gradient elution (0-2min, 100-70% A; 2-12min, 70-30% A; 12-12.5 min, 30-5% A; 12.5-13min, 5-0% A; 13-14min, 0-0% A; 14- 14.1 min, 0-100% A; 14.1-16 min, 100-100% A). The flow rate of the mobile phase was 0.26mL/min. The column temperature was maintained at 40°C. The injection volume was 3μl. The mass spectrometer was used in the positive electrospray ionization mode. The pos spray voltages were 5500V, and the turbospray temperature was 500 °C. Analysis software was used for systemic control and data acquisition.

Table S1 Comparison of 39 sphingolipids between G<2 and G≥2 in 70 CHB patients at baseline.

Variables	G<2 (n=27)	G≥2(n=43)	*P* value
SM d16:0/16:1	4.95±1.29	5.72±1.49	0.027
SM d16:1/16:1	0.41±0.13	0.46±0.13	0.091
SM d16:0/17:1	3.81±0.95	4.21±1.11	0.075
SM d18:1/15:1	0.44±0.19	0.50±0.19	0.093
SM d16:0/18:0	10.39±3.42	10.89±3.53	0.409
SM d16:0/18:1	148.22±33.38	144.55±33.20	0.947
SM d16:1/18:1	17.66±4.58	17.03±3.72	0.856
SM d16:0/18:3	0.09±0.34	0.10±0.04	0.712
SM d18:0/17:0	2.06±0.71	2.23±0.79	0.320
SM d16:0/19:1	4.35±1.28	4.55±1.61	0.579
SM d18:1/17:1	0.40±0.10	0.39±0.12	0.777
SM d16:0/20:1	32.33±9.74	31.51±10.74	0.880
SM d18:0/18:2	13.79±3.86	12.20±3.73	0.136
SM d16:0/20:3	0.66±0.18	0.59±0.19	0.141
SM d18:1/19:0	4.14±1.32	4.18±1.17	0.571
SM d18:1/19:1	9.55±3.92	9.63±3.84	0.772
SM d16:0/22:1	20.94±4.72	21.15±6.23	0.976
SM d18:1/20:1	15.57±4.72	14.41±4.67	0.530
SM d16:0/22:3	0.85±0.22	0.87±0.30	0.530
SM d20:0/19:0	1.07±0.25	1.10±0.29	0.412
SM d20:0/19:1	4.73±1.21	5.11±1.61	0.239
SM d16:1/24:0	51.31±14.19	47.91±16.35	0.337
SM d16:1/24:1	34.07±11.48	32.91±9.56	0.488
SM d18:1/22:2	6.83±2.18	7.70±2.52	0.139
SM d16:1/25:0	16.55±4.91	17.11±5.79	0.713
SM d16:0/26:1	39.32±12.59	37.21±13.16	0.436
SM d18:1/24:1	69.17±29.23	62.16±21.94	0.444
SM d20:0/22:4	52.29±17.51	53.70±17.52	0.617
SM d20:0/22:5	58.90±19.08	58.11±22.79	0.554
SM d20:0/22:6	0.72±0.27	0.69±0.23	0.558
SM d18:1/25:0	1.41±0.43	1.49±0.60	0.781
SM d18:2/25:0	1.44±0.67	1.69±0.90	0.320
Cer d18:1/18:0	0.22±0.09	0.21±0.08	0.629
Cer d18:1/20:0	0.25±0.09	0.22±0.07	0.174
Cer d18:1/22:0	0.19±0.09	0.19±0.06	0.251
Cer d18:2/22:0	0.31±0.14	0.24±0.09	0.021
Cer d18:1/24:1	1.42±0.67	1.21±0.40	0.235
Cer d18:2/24:1	0.26±0.14	0.19±0.07	0.055
Cer d18:1/25:0	3.44±0.24	3.46±0.16	0.242

Table S2 Comparison of 35 sphingolipids between G<2 and G≥2 in 126 cases

Variables	G<2 (n=80)	G≥2 (n=46)	*P* value
SM d16:1/16:1 (μmol/L), SD	0.39±0.14	0.45±0.13	0.013
SM d18:1/15:1 (μmol/L), SD	0.43±0.19	0.49±0.19	0.028
SM d16:0/18:0 (μmol/L), SD	9.22±3.22	10.54±3.66	0.029
SM d16:0/18:1 (μmol/L), SD	138.39±34.25	141.54±34.11	0.423
SM d16:1/18:1 (μmol/L), SD	17.23±4.18	16.72±3.82	0.700
SM d16:0/18:3 (μmol/L), SD	0.09±0.04	0.09±0.04	0.588
SM d16:0/19:1 (μmol/L), SD	4.05±1.39	4.42±1.65	0.184
SM d18:1/17:1 (μmol/L), SD	0.40±0.11	0.38±0.12	0.616
SM d16:0/20:1 (μmol/L), SD	29.24±10.51	30.65±10.95	0.412
SM d18:0/18:2 (μmol/L), SD	13.15±3.96	11.94±3.81	0.196
SM d16:0/20:3 (μmol/L), SD	0.66±0.18	0.58±0.20	0.020
SM d18:1/19:0 (μmol/L), SD	3.69±1.22	4.07±1.21	0.051
SM d18:1/19:1 (μmol/L), SD	8.40±3.39	9.31±3.91	0.211
SM d16:0/22:1 (μmol/L), SD	19.75±5.49	20.84±6.14	0.347
SM d18:1/20:1 (μmol/L), SD	14.68±4.93	14.11±4.75	0.750
SM d16:0/22:3 (μmol/L), SD	0.79±0.28	0.85±0.31	0.248
SM d20:0/19:0 (μmol/L), SD	0.99±0.27	1.08±0.30	0.072
SM d20:0/19:1 (μmol/L), SD	4.74±1.33	5.04±1.58	0.254
SM d16:1/24:0 (μmol/L), SD	50.07±14.62	47.31±16.04	0.244
SM d16:1/24:1 (μmol/L), SD	34.82±10.65	32.35±9.49	0.153
SM d18:1/22:2 (μmol/L), SD	6.12±2.30	7.48±2.61	0.010
SM d16:1/25:0 (μmol/L), SD	16.31±4.98	16.87±5.67	0.678
SM d16:0/26:1 (μmol/L), SD	38.02±11.76	36.67±12.98	0.354
SM d18:1/24:1 (μmol/L), SD	61.62±29.95	60.81±21.90	0.536
SM d20:0/22:4 (μmol/L), SD	49.38±17.83	52.85±18.25	0.267
SM d20:0/22:5 (μmol/L), SD	54.95±19.03	57.85±22.26	0.663
SM d20:0/22:6 (μmol/L), SD	0.71±0.27	0.68±0.25	0.605
SM d18:1/25:0 (μmol/L), SD	1.28±0.46	1.45±0.60	0.191
SM d18:2/25:0 (μmol/L), SD	1.29±0.68	1.64±0.90	0.032
Cer d18:1/18:0 (μmol/L), SD	0.20±0.08	0.21±0.08	0.637
Cer d18:1/20:0 (μmol/L), SD	0.22±0.08	0.22±0.07	0.966
Cer d18:1/22:0 (μmol/L), SD	0.19±0.08	0.19±0.06	0.438
Cer d18:1/24:1 (μmol/L), SD	1.27±0.57	1.20±0.42	0.831
Cer d18:2/24:1 (μmol/L), SD	0.24±0.11	0.19±0.07	0.020
Cer d18:1/25:0 (μmol/L), SD	3.46±0.22	3.47±0.16	0.397

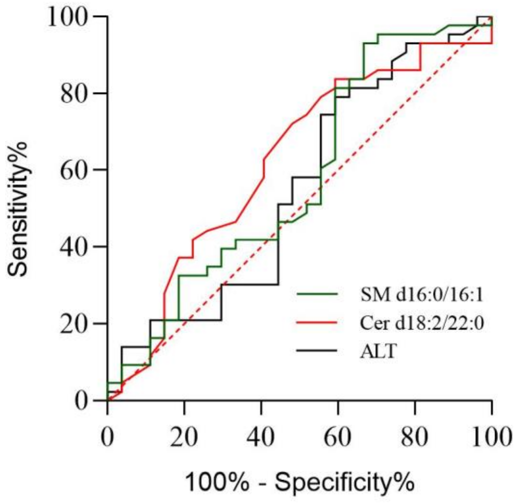

Figure S1 The ROC analysis of ALT, SM d16:0/16:1 and Cer d18:2/22:0 for the diagnosis of significance necroinflammation (G ≥ 2).
